# Supplementary material for: The prospective COVID-19 vaccine: willingness to pay and perception of community members in Ibadan, Nigeria
Source: PeerJ. 2021 Mar 26;9:e11153. doi: 10.7717/peerj.11153 (PMC8006750; doi:10.7717/peerj.11153)
Supplement: Supplemental Information 2 [file peerj-09-11153-s002.docx]

| **CODE BOOK** | | | | | | | | | | | | | | | | | | |
| --- | --- | --- | --- | --- | --- | --- | --- | --- | --- | --- | --- | --- | --- | --- | --- | --- | --- | --- |
| **Sociodemographic characteristics** | | | | | | | | | | | | | | | | | | |
| **1.Age** *(at last birthday):* |  | | |  | | | | | |  | |  | | | | | |  |
| **2. Sex** | 1 Male | | | | | | 2 Female | | | | | | | | | |  |  |
| **3. Occupation:** |  | | | | | | | | | | | | | | | | |  |
| **4. Religion:** | 1 Christian | | | | | | | 2 Islam | | | | 3 Traditional | | | | 4 Others | |  |
| **5. Highest level of education:** | 1 None | | | | | | | 2 Primary | | | | 3 Secondary | | | | 4 Tertiary | |  |
| **6. Ethnic group** | 1. Yoruba | | | | 2. Igbo | | | | | 3. Hausa | | 4. Other (specify) ……… | | | | | |  |
| **7. Marital status** | 1. Married | | | | 2. Single | | | | | 3. Widowed | | 4. Divorced | | | | | |  |
| **8. Average monthly income** | ______________________________ | | | | | | | | | | | | | | | | |  |
| **9. Do you own the house that you live in?** | | | 0=No | | | | | | | | | | | **1. Yes** | | | |  |
| **10. Which of the following items do you have in your house? (the interviewer should mention the items one after the other)** | | | | | | | | | | | | | | | | | |  |
| \| Items \| Absent \| Present \|  \| Items \| Absent \| Present \| \| --- \| --- \| --- \| --- \| --- \| --- \| --- \| \| 1. Stove \| 0 \| 1 \|  \| 8. Piped water in your household \| 0 \| 1 \| \| 2. Fan \| 0 \| 1 \|  \| 9 .Bicycle \| 0 \| 1 \| \| 3. Refrigerator \| 0 \| 1 \|  \| 10. Motorcycle \| 0 \| 1 \| \| 4. Air Conditioner \| 0 \| 1 \|  \| 11. Motor vehicle \| 0 \| 1 \| \| 5. Radio \| 0 \| 1 \|  \| 12. Upholstered chairs \| 0 \| 1 \| \| 6. Television \| 0 \| 1 \|  \| 13. Sewing machine \| 0 \| 1 \| \| 7. Generator \| 0 \| 1 \|  \| 14. Washing machine \| 0 \| 1 \| | | | | | | | | | | | | | | | | | |  |
| **Knowledge of COVID-19** | | | | | | | | | | | | | | | | | | |
| **11. Have you ever heard about COVID-19?** | | | | | | | | | 1 Yes | | | | | | 2 No | | | |
| **12. What causes COVID-19? *(****Do not read options, tick what is mentioned. Multiple responses allowed)* | | | | | | | | | | | | | | | | | | |
| 1. Contact with saliva from a person who is sick with COVID-19 2. Participating in burial rites of a person who has died from COVID-19   6. Other ways (pls specify) | | | | | | 3. Contact with beddings, clothing and other personal utensils (plates, cups) of a person who is sick of COVID-19  4. Mosquito bites  5. Respiratory droplets of an infected persons | | | | | | | | | | | | |
| **Knowledge of COVID-19 vaccine**  **13. Have you heard of the prospective COVID-19 vaccine?** 1. Yes 2. No  **14. If yes, what are your sources of information?** ***(Do not read options, tick what is mentioned. Multiple responses allowed)***  1. Radio 2. Television 3. Journal 4. Newspaper 5. Health Educator 6. Town announcer  7. Mosque 8. Church 9. Family member 10. Peers 11. Health facility 12. Flyer 13. Internet sites  14. Social media (Facebook, Twitter, WhatsApp) 15. GSM/SMS 16. Neighborhood 17. Others (pls state) | | | | | | | | | | | | | | | | | | |
| **Perceptions about COVID-19 vaccine (tick one SA-Strongly agree, A-Agree, N-not decided, D-Disagree, SD-Strongly disagree** | | | | | | | | | | | | | | | | | | |
| \| **SN** \| **Perception questions** \| **SA** \| **A** \| **N** \| **D** \| **SD** \| \| --- \| --- \| --- \| --- \| --- \| --- \| --- \| \| **15** \| **COVID-19 is a major public health problem requiring vaccine** \| **5** \| **4** \| **3** \| **2** \| **1** \| \| **16** \| **COVID-19 vaccine will prevent COVID-19** \| **5** \| **4** \| **3** \| **2** \| **1** \| \| **17** \| **COVID-19 vaccine should get administered to everyone** \| **5** \| **4** \| **3** \| **2** \| **1** \| \| **18** \| **COVID-19 vaccine is against our cultural belief** \| **1** \| **2** \| **3** \| **4** \| **5** \| \| **19** \| **COVID-19 vaccine will save productive hours lost to COVID-19 illness** \| **5** \| **4** \| **3** \| **2** \| **1** \| \| **20** \| **COVID-19 vaccine will save money spent on COVID-19 treatment** \| **5** \| **4** \| **3** \| **2** \| **1** \| \| **21** \| **I will take the COVID-19 vaccine when produced** \| **5** \| **4** \| **3** \| **2** \| **1** \| \| **22** \| **COVID-19 vaccine will not have adverse health effect** \| **5** \| **4** \| **3** \| **2** \| **1** \| | | | | | | | | | | | | | | | | | | |
| **Willingness to pay for COVID-19 vaccine** | | | | | | | | | | | | | | | | | | |
| **23. Are you willing to pay for the COVID-19 vaccine?** | | | | | | | | | | | | | | | | | | |
| 1. Yes | | 1. No | | | | | | | | | | | 1. I don’t know | | | | | |
| **24. What maximum amount are you willing to pay for the vaccine? *(Read options)*** 1. #45000 2. #25000 3. Other  (*pls specify) ……………………*  **25. If yes to Q23, specify reasons for your willingness** ***(Do not read options, tick what is mentioned. Multiple responses allowed)*** | | | | | | | | | | | | | | | | | | |
| 1. To stay healthy | | | | | | 2. To prevent loss of productive hours | | | | | | | | | | | | |
| 3. To prevent further treatment expenses | | | | | | 4. To promote social acceptability of COVID-19 vaccines | | | | | | | | | | | | |
| 5. Other *(please state) …………………………………………………………………………………………………………………* | | | | | | | | | | | | | | | | | | |
| **26. If no to Q*23*, specify *(Do not read options, tick what is mentioned. Multiple responses allowed)*** | | | | | | | | | | | | | | | | | | |
| 1. Costs not affordable by households | | 1. Fear of adverse effects | | | | | | | | | 1. Fear of inaccessibility of vaccine | | | | | | | |
| 1. Contrary to religious beliefs | | 1. Contrary to culture | | | | | | | | |  | | | | | | | |
| 1. Other *(please state)* | | | | | | | | | | | | | | | | | | |
| **Intent to comply with the prospective COVID-19 vaccine** | | | | | | | | | | | | | | | | | | |
| **27. Would you allow members of your household to take the COVID-19 vaccine?** 1. Yes 2. No 3. Don’t know  **28. If no to *27*, specify reasons..………………………………………………………………………………………………………………………** | | | | | | | | | | | | | | | | | | |
| **29. Would you require any specific information on the COVID-19 vaccine before accepting the vaccine?**1. Yes 2. No  **30. If yes to *29*, specify *(Do not read options, tick what is mentioned. Multiple responses allowed)***   1. Whether the vaccine would prevent or cure COVID-19 2. Number of doses needed 3. Route of administration 4. Age range of individuals to be vaccinated 5. Vaccine collection points 6. Manufacturer of the vaccine 7. Whether payments would be required 8. Whether vaccination would be accompanied by incentives 9. Side effects of the vaccine 10. Others (please state) | | | | | | | | | | | | | | | | | | |
