# Supplementary material for: The prospective COVID-19 vaccine: willingness to pay and perception of community members in Ibadan, Nigeria
Source: PeerJ. 2021 Mar 26;9:e11153. doi: 10.7717/peerj.11153 (PMC8006750; doi:10.7717/peerj.11153)
Supplement: Supplemental Information 3 [file peerj-09-11153-s003.pdf]

Informed Consent form

Prospective COVID-19 Vaccine: Knowledge, and Perception of community members in Oyo State

Dear respondent,
My name is ____. I am part of a group of researchers from the University of Ibadan. In response to the outbreak of COVID-19 in Nigeria and as part of the government's effort to curtail the spread of the disease, we are conducting this study to assess the knowledge, attitude, and practices of community members regarding COVID-19 vaccine in Nigeria. The result of the study will be used to guide the development and dissemination of appropriate public health messages that will address knowledge gaps, correct misconception, and improve practices that will improve the uptake of COVID-19 vaccine when available.
We kindly request you to respond to the following questions to the best of your knowledge. Be assured that all your responses will be treated with utmost confidentiality. Your participation in the study will be highly appreciated.
You are free to refuse to take part in the study. You have the right to withdraw at any given time if you choose to.
Now that the study has been well explained to you and you fully understand the content of the process, are you willing to take part in the study?
1. Yes 2. No
If no give reasons ____
Signature/ thumbprint of participant………………….. Interview date ……………………

Questionnaire

Prospective COVID-19 Vaccine: Knowledge, and Perception of community members in Oyo State

Name of Interviewer<br>LGA<br>Name of street			Serial Number<br>Date of interview *(dd/mm/yyyy)*	
**Sociodemographic characteristics**				
**1.Age** *(at last birthday):*				
**2. Sex**	1 Male		2 Female	
**3. Occupation:**				
**4. Religion:**	1 Christian	2 Islam	3 Traditional	4 Others
**5. Highest level of education:**	1 None	2 Primary	3 Secondary	4 Tertiary
**6. Ethnic group**	1. Yoruba	2. Igbo	3. Hausa	4. Other (specify) ………
**7. Marital status**	1. Married	2. Single	3. Widowed	4. Divorced
**8. Average monthly income**	______________________________			
**9. Do you own the house that you live in?**	**1. Yes**		**2. No**	
**10. Which of the following items do you have in your house? (the interviewer should mention the items one after the other)**				

Items	Present	Items	Present
1. Stove		8. Piped water in your household	
2. Fan		9 .Bicycle	
3. Refrigerator		10. Motorcycle	
4. Air Conditioner		11. Motor vehicle	
5. Radio		12. Upholstered chairs	
6. Television		13. Sewing machine	
7. Generator		14. Washing machine	

**Knowledge of COVID-19**		
**11. Have you ever heard about COVID-19?**	1 Yes	2 No

12. What causes COVID-19? *(Do not read options, tick what is mentioned. Multiple responses allowed)*
1. Contact with saliva from a person who is sick with COVID-19
2. Participating in burial rites of a person who has died from COVID-19
3. Contact with beddings, clothing and other personal utensils (plates, cups) of a person who is sick of COVID-19
4. Mosquito bites
5. Respiratory droplets of an infected persons
6. Other ways (pls specify)

Knowledge of COVID-19 vaccine

13. Have you heard of the prospective COVID-19 vaccine? 1. Yes 2. No

14. If yes, what are your sources of information? ***(Do not read options, tick what is mentioned. Multiple responses allowed)***

1. Radio 2. Television 3. Journal 4. Newspaper 5. Health Educator 6. Town announcer
7. Mosque 8. Church 9. Family member 10. Peers 11. Health facility 12. Flyer 13. Internet sites
14. Social media (Facebook, Twitter, WhatsApp) 15. GSM/SMS 16. Neighborhood 17. Others (pls state)

Perceptions about COVID-19 vaccine (tick one SA-Strongly agree, A-Agree, N-not decided, D-Disagree, SD-Strongly disagree

SN	Perception questions	SA	A	N	D	SD
15	**COVID-19 is a major public health problem requiring vaccine**					
16	**COVID-19 vaccine will prevent COVID-19**					
17	**COVID-19 vaccine should get administered to everyone**					
18	**COVID-19 vaccine is against our cultural belief**					
19	**COVID-19 vaccine will save productive hours lost to COVID-19 illness**					
20	**COVID-19 vaccine will save money spent on COVID-19 treatment**					
21	**I will take the COVID-19 vaccine when produced**					
22	**COVID-19 vaccine will not have adverse health effect**					

Willingness to pay for COVID-19 vaccine

23. Are you willing to pay for the COVID-19 vaccine?

1. Yes 2. No 3. I don't know

24. What maximum amount are you willing to pay for the vaccine? ***(Read options)*** 1. #45000 2. #25000 3. Other *(pls specify)* ……………………

25. If yes to Q23, specify reasons for your willingness ***(Do not read options, tick what is mentioned. Multiple responses allowed)***

1. To stay healthy
2. To prevent loss of productive hours
3. To prevent further treatment expenses
4. To promote social acceptability of COVID-19 vaccines
5. Other *(please state)* ……………………………………………………………………………………………

26. If no to Q*23*, specify ***(Do not read options, tick what is mentioned. Multiple responses allowed)***

1. Costs not affordable by households
2. Fear of adverse effects
3. Fear of inaccessibility of vaccine
4. Contrary to religious beliefs
5. Contrary to culture
6. Other *(please state)*

Intent to comply with the prospective COVID-19 vaccine

27. Would you allow members of your household to take the COVID-19 vaccine? 1. Yes 2. No 3. Don't know

28. If no to *27*, specify reasons..……………………………………………………………………………………………………

29. Would you require any specific information on the COVID-19 vaccine before accepting the vaccine?1. Yes 2. No

30. If yes to *29*, specify ***(Do not read options, tick what is mentioned. Multiple responses allowed)***

1. Whether the vaccine would prevent or cure COVID-19
2. Number of doses needed
3. Route of administration
4. Age range of individuals to be vaccinated
5. Vaccine collection points
6. Manufacturer of the vaccine
7. Whether payments would be required
8. Whether vaccination would be accompanied by incentives
9. Side effects of the vaccine
10. Others (please state)

Thank you
